# Development of a person-centred care approach for persons with chronic multimorbidity in general practice by means of participatory action research

**DOI:** 10.1186/s12875-024-02364-x

**Published:** 2024-04-16

**Authors:** Mieke JL Bogerd, Pauline Slottje, Jettie Bont, Hein PJ Van Hout

**Affiliations:** 1 Amsterdam UMC, location VUmc, Department of General Practice, Boelelaan 1117, Amsterdam, The Netherlands; 2 Amsterdam Public Health, Quality of Care, Amsterdam, The Netherlands; 3 Amsterdam UMC, location AMC, Department of General Practice, Meibergdreef 9, Amsterdam, the Netherlands; 4 Amsterdam Public Health, Aging & Later life, Amsterdam, the Netherlands

**Keywords:** Chronic diseases, General Practice, Multimorbidity, person-centred care, Participatory action research

## Abstract

**Background:**

The management of persons with multimorbidity challenges healthcare systems tailored to individual diseases. A person-centred care approach is advocated, in particular for persons with multimorbidity. The aim of this study was to describe the co-creation and piloting of a proactive, person-centred chronic care approach for persons with multimorbidity in general practice, including facilitators and challenges for successful implementation.

**Methods:**

A participatory action research (PAR) approach was applied in 13 general practices employing four subsequent co-creation cycles between 2019 and 2021. The target population included adults with ≥3 chronic conditions. Participating actors were general practitioners (GPs), practice nurses (PNs), patients (target group), the affiliated care cooperation, representatives of a health insurer and researchers. Each cycle consisted of a try-out period in practice and a reflective evaluation through focus groups with healthcare providers, interviews with patients and analyses of routine care data. In each cycle, facilitators, challenges and follow-up actions for the next cycle were identified. Work satisfaction among GPs and PNs was measured pre and at the end of the final co-creation cycle.

**Results:**

Identified essential steps in the person-centred chronic care approach include (1) appropriate patient selection for (2) an extended person-centred consultation, and (3) personalised goalsetting and follow-up. Key facilitators included improved therapeutic relationships, enhanced work satisfaction for care providers, and patient appreciation of extended time with their GP. Deliberate task division and collaboration between GPs and PNs based on patient, local setting, and care personnel is required. Challenges and facilitators for implementation encompassed a prioritisation tool to support GPs appropriately who to invite first for extended consultations, appropriate remuneration and time to conduct extended consultations, training in delivering person-centred chronic care available for all general practice care providers and an electronic medical record system accommodating comprehensive information registration.

**Conclusions:**

A person-centred chronic care approach targeting patients with multimorbidity in general practice was developed and piloted in co-creation with stakeholders. More consultation time facilitated better understanding of persons’ situations, their functioning, priorities and dilemma’s, and positively impacted work satisfaction of care providers. Challenges need to be tackled before widespread implementation. Future evaluation on the quadruple aims is recommended.

**Supplementary Information:**

The online version contains supplementary material available at 10.1186/s12875-024-02364-x.

## Background

A growing number of patients with multimorbidity are inappropriately and inefficiently served through traditional single disease care programmes in general practice [1–3]. Multimorbidity, defined as the coexistence of multiple chronic conditions in one individual [4], can have a major impact on persons’ lives [5]. Compared to persons with a single chronic disease, persons with multimorbidity have a lower life expectancy, are more likely to be admitted to a hospital, have a poorer quality of life and are at increased risk of polypharmacy-associated adverse events and difficulties with adherence [6, 7]. People with multimorbidity often experience problems in multiple areas (e.g. physical, psychological and social) [2] and often multiple care professionals are involved, making it challenging to achieve a comprehensive overview and coordinated care management [8, 9].

In the Netherlands as well as in other European countries, single disease management programmes (DMPs) in general practice have been developed to improve care for persons with certain highly prevalent chronic conditions. These programmes have been shown to improve lifestyle (e.g. physical activity) and short term health indicators (e.g. blood sugar levels) [10]. However, in the case of multimorbidity, managing each disease separately may lead to fragmented care, confusion in patients due to contradictory or complex medication and lifestyle regimens, and adverse health outcomes [6, 7]. Also, scheduled standard check-up appointments instead of individually tailored care, may be inefficient and time-consuming for patients as well as for care professionals. A shift from a disease-specific to a more person-centred chronic care approach is proposed [2] to overcome these disadvantages and to acknowledge and take into account personal goals in life and care preferences, as well as social, cognitive and emotional circumstances. When appropriately developed and implemented, person-centred care is expected to contribute to the quadruple aims: enhancing patient experience, improving patients’ health outcomes, improving the work experience of healthcare professionals and reducing costs of resource use [11]. However, the evidence on the potential benefits and risks of such a person-centred care approach is still limited and inconclusive. A Cochrane systematic review of person-centred initiatives in primary care and community settings reported improved mental health outcomes and improved healthcare provider behaviour, yet inconclusive effects on participants’ physical health [12]. A pragmatic cluster-randomised trial in general practice conducted by Salisbury et al., found no significant effects of 6-monthly comprehensive 3D reviews incorporating patient-centred strategies, on multimorbid patients' health-related quality of life [13].

In 2021, the UK Medical Research Council Guidance introduced a framework for the development and evaluation of complex interventions, such as a new person-centred chronic care approach for patients with multimorbidity [14]. The framework advocates that complex interventions should be carefully developed and refined with sufficient consideration of local context, meaningful stakeholder engagement, early implementation planning, and small-scale piloting, for example using participatory action research (PAR) [15, 16].

The objective of the current PAR project was to describe the co-creation and pilot test the of a proactive, person-centred chronic care approach in general practice for patients with multimorbidity, and to elucidate factors relevant for successful implementation.

## Methods

### Study design

A participatory action research study was conducted, consisting of four plan-act-observe-reflect-adjust-cycles. The pilot test primarily focused on the process.

#### Setting

In the Netherlands, all community dwelling citizens are registered with one general practice. A general practice serves on average 2095 persons [17]. The GP functions as the first point of medical care and as gatekeeper for specialised care. Free access to primary care is guaranteed. In the Netherlands, care cooperations take on organisational responsibilities, enabling individual general practices to facilitate protocolised DMPs for chronic conditions in a particular region [18], namely for diabetes mellitus (type 2), Chronic Obstructive Pulmonary Disease and Cardiovascular Risk Management. Practice nurses carry out the majority of the DMPs’ according to care protocols.

This study was built on ideas generated in a think tank with prominent GPs in the North-West region of the Netherlands. The thirteen participating general practices collaborate within the primary care cooperation ‘Huisartsen Zuid-Kennemerland (HZK)’. To promote thinking outside current financial constraints during the co-creation process, a distinction was made between financial and substantive aspects of the person-centred approach. Here, we focus on the latter.

#### Co-creation team

Participating actors were GPs, PNs, patients from the target group, the affiliated care cooperation, representatives of a health insurer and researchers. A co-creation team was formed and consisted of three GPs, one representative of the care cooperation, four researchers from the department of general practice at Amsterdam University Medical Center and one external project leader. After the second cycle, two representatives of practice nurses (PNs) were added to the co-creation team. Over the course of the project, biannual meetings with the health insurer were organised to discuss progress and exchange of ideas (‘sparring partners’). Supplementary Figure 1 illustrates the steering and decision making moments including the stakeholder groups involved during the co-creation process. To incorporate the patient perspective, the research team integrated the results of patient interviews into the co-creation process in each cycle, with a minimum of five interviews conducted per research cycle.

As customary in PAR, the researchers acted both as participants and facilitators [15]. In this study, a PhD candidate who was also a GP trainee and had a master’s degree in healthcare management facilitated the PAR process under the supervision of four senior researchers, including two GPs. Their expertise covered primary and elderly care, medical education, epidemiology, routine general practice care data research, and PAR.

### ‘Extended person-centred consultation’ as the starting point

The idea of a pro-active ‘extended person-centred consultation’ (EPCC) came from Think tank GPs as the starting point for the first research cycle [19]. This consultation (performed by GPs) took between 30 and 60 minutes instead of the regular 10-min consultation. Per consultation, a fee of 63 euro could be claimed as reimbursement. Health status, healthcare needs, personal goals and the patients’ context and preferences were to be discussed. To provide some structure during the EPCC, a model for shared decision-making on goals and care arrangements (NHG, 2017) was offered to all participating GPs. Person-centred goalsetting was recognised as a key element in the person-centred approach, viewing it as a potential means to tailor follow-up on an individual basis. A goalsetting procedure was employed to monitor the extent to which patients achieved their individual goals. In addition, follow-up of patients after the consultation was tailored to the goals that were set. Other disciplines (e.g., PNs, physiotherapists and psychologists) could be involved.

### Recruitment

#### General practices

Thirteen general practices situated in the North-West region of the Netherlands, that were associated with the same care cooperation participated in the study, representing a total of 22 GPs, 11 PNs and 4090 patients with multimorbidity. The age range of patients spanned from 18 to 99 years, with 67% being aged 65 years or older. The majority of practices had participated in the exploratory phase (think tank) preceding this project. The rest were recruited via the care cooperation’s digital newsletter. All (but one GP) were partner GPs and worked at their practice for more than 10 years. Practice types included single-handed, duo and group practices. Two practices served patients with a predominantly low socioeconomic status. In the other practices, socioeconomic status ranged from low to high.

#### Target population

The ultimate goal was to develop a chronic care approach applicable to all patients with a chronic disease in general practice. The co-creation team decided to focus on those with multimorbidity first as they were expected to benefit most from person-centred chronic care. Multimorbidity was defined as having at least three chronic conditions from a predefined list (Supplementary Table 1), encompassing specific risk factors (e.g. hypertension, hypercholesterolemia, tobacco or alcohol abuse). The diagnoses are registered by Dutch GPs using the International Classification of Primary Care (ICPC). Patients were excluded when they were terminally ill, were diagnosed with dementia (ICPC P70) or mental retardation/intellectual disability (ICPC P85), or were severely hearing of visual impaired (ICPC H86 or F94). Also, patients were excluded when they were enrolled in a care programme for vulnerable elderly persons.

#### Patient selection procedure

A uniform search string based on electronic medical record (EMR) data was developed and applied by GPs to list potentially eligible multimorbid patients for an EPCC [19]. GPs were allowed to select patients and checked if the preselected patients met the selection criteria based on their professional discretion. Furthermore, the search string enabled the GP to stratify these preselected multimorbid patients according to predefined strata based on two criteria. First, whether or not patients had at least one chronic condition that made them eligible for a DMP. Second, whether the contact frequency with the general practice in the past two years was considered to be low (less than 20), medium (20–40) or high (more than 40 contacts) [20]. As described below, to ensure healthcare providers had the opportunity to gain experience with a diverse group of multimorbid patients, preselected subgroup of the (stratified) target group was provided for them to choose from and invite for an EPCC. The co-creation group deliberately entrusted the responsibility of patient selection to healthcare providers, given their pivotal role in the development process. As the primary aim of the research was developmental rather than evaluative, sample selection bias was not deemed a substantial obstacle.

### Data collection and decision-making process

#### Research cycles

In this study, an initial exploratory phase established the starting point, followed by four iterative plan-act-observe-reflect-adjust cycles lasting approximately six months each. Each cycle comprised a four-month 'action phase' and a two-month 'evaluation and adjust phase'. Participants (GPs and PNs) actively gained experience with the person-centred chronic care approach by conducting five to ten EPCCs per cycle during the 'action phase'. The subsequent 'evaluation phase' involved reflection on the actions taken. Data collection and analysis involved extracting pseudonymised routine care data from the EMR, conducting focus groups with healthcare providers (led by an experienced moderator, HvH) and patient interviews (thirteen by MB, eight by MN). This consisted of a random sample of all patients who underwent an EPCC, taking into account representation of all practices. Interviews covered various aspects, such as EPCC experiences, positive and negative feedback, follow-up agreements, person-centred goalsetting, essential consultation elements, and potential improvements. The researchers processed all gathered information through thematic interpretation of the qualitative material and by generating descriptive overviews based on the EMR. These overviews provided insight into patient demographics, conditions, contact frequency with general practice, and patient subgroup identification. Throughout each cycle, the research team held sessions to discuss and interpret data, leading to collaborative identification of challenges, facilitators, and potential follow-up actions for the next cycle. These findings were presented and discussed with the co-creation team, leading to a collectively formulated adjusted approach for the subsequent cycle. This adjusted approach was transparently communicated to all participating care professionals, who actively proposed and decided upon adjustments to be tested in the next cycle. Data collection occurred between January 2019 and June 2021, with some focus groups, interviews, and research meetings conducted online due to the COVID-19 pandemic.

#### Person-centred goalsetting

To gain more insight into the person-centred goalsetting process, all anonymised (free text) journal reports were extracted from the EMR after the last research cycle. These journal reports were flagged by care professionals using the ICPC-code A58 (= Therapeutic conversation/counselling) and ‘COPILOT’. To ensure that all journals were found the search query was expanded with specific search terms (e.g. person-centred consultation). In the second cycle, GPs were asked to review journal reports of previously registered EPCCs (henceforth referred to as person-centred goalsetting assignments). They were asked to report the agreed person-centred goals, describe barriers and facilitators to setting person-centred goals.

#### Work satisfaction of care professionals

Work satisfaction among GPs and PNs was measured pre and at the end of the final co-creation cycle.

Existing questionnaires did not capture work satisfaction specifically on caring for persons with multimorbidity. Therefore, the researchers developed a topic list concerning job satisfaction and experienced regulatory burden in close collaboration with the initiating GPs. Based on these topics, twelve statements were formulated, i.e. “I derive satisfaction in my work activities related to providing chronic care to individuals with multiple chronic diseases.” (see Supplementary Box 1). Response options range from 0 (agree) to 10 (totally disagree). The questionnaire was pilot tested and refined in the preparation phase [19].

In total, 15 recorded focus groups (11 with GPs, 4 with PNs), interviews with GPs (and PN if available) of 10 different participating general practices to create a practice profile (e.g. organisation, staff, vision), 21 semi-structured patient interviews, 4 co-creation team meetings, ten ‘person-centred goal setting’ assignments as reported by GPs and all minutes of weekly researcher meetings (over 2.5 years) that were used in the decision-making process of the pilot, were collected.

### Data analysis

Summaries of all collected data were thematically analysed [21]. If the summaries were unclear to the researchers, the original minutes or audiotape recordings of meetings were analysed. The initial two focus groups underwent independent coding by two researchers (MB, MN), and the results were deliberated to evaluate consensus and enhance the code scheme. Because of consistency in coding and the substantial amount of available data, the rest of the subsequent analyses were performed by one researcher (MB). In discussions with all involved researchers, categories were formulated, refined and reduced into major themes. Within these themes, facilitators, challenges and required actions were identified.

A thematic analysis was also performed on the EMR journal reports of all extracted EPCCs. The journal reports were coded, based on problems noted and formulated goals/agreements. The first ten journal reports were independently coded by two researchers (MB, CvH) (and the results were discussed to reach consensus and improve the codes scheme. Due to consistency in coding between the two researchers, the rest of the data were coded and analysed by one researcher with a second researcher verifying the codes. 

Regarding the work satisfaction questionnaires, pre and post-study results were compared using paired T-test analysis at item level.

## Results

**Fig. 1:**
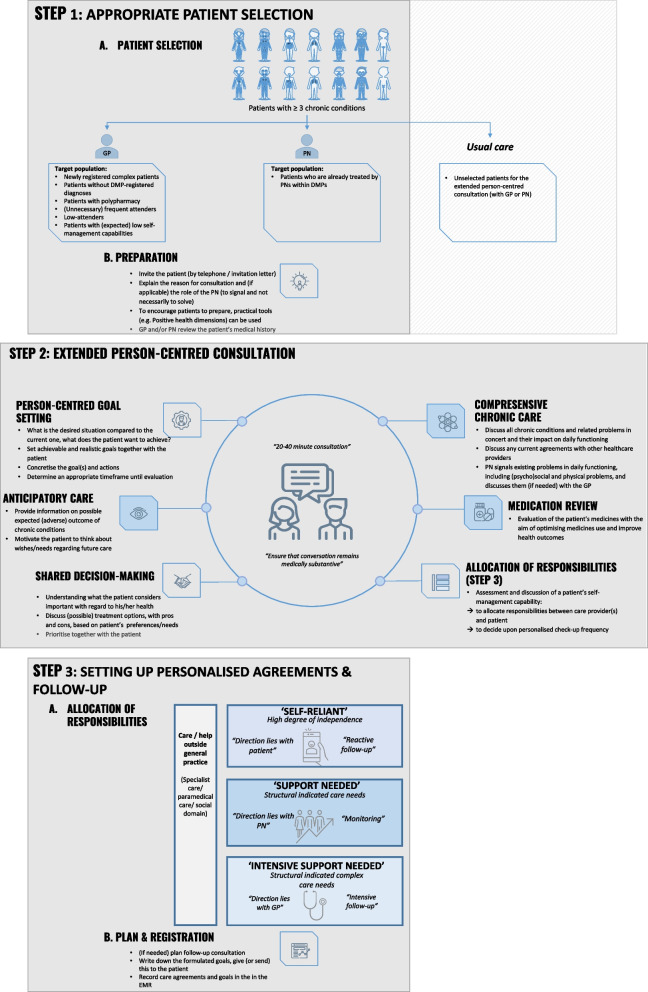
Visualisation of co-created person-centred chronic care approach (three steps) for multimorbid patients in general practice.

The final person-centred chronic care approach that was co-created during the four research cycles is illustrated in Fig. 1. The central element of the approach was the EPCC. This element was piloted and iterated upon, aiming to identify the appropriate patient selection and determine who should conduct the EPCC (GP and/or PN) (Step 1), specifying the content of the EPCC (Step 2), and setting up personalised agreements and follow-up (Step 3).

**Fig. 2:**
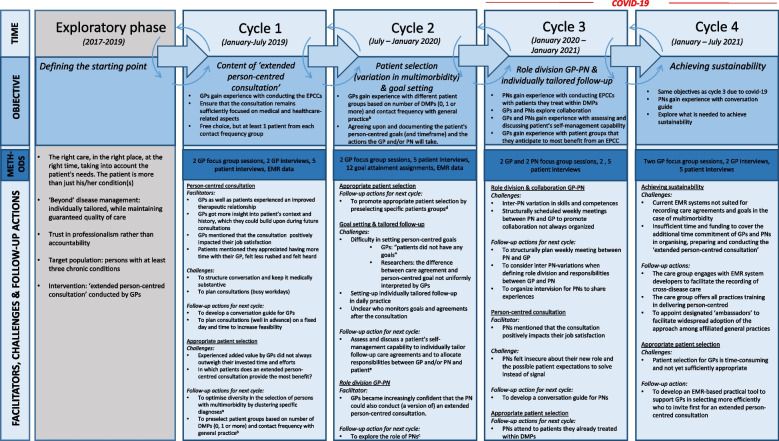
Visualisation of the development process including one exploratory phase and four act-observe-reflect-adjust co-creation cycles. **A **To optimise diversity in the selection of persons with multimorbidity, GPs suggested to cluster certain chronic conditions, such as cardiovascular risks and diseases, asthma and COPD, and several osteoarthritis conditions. **B** GPs generated a patient list in which patients were grouped based on information extracted from the EMR by means of uniform queries developed for this project. **C** i.e. joint EPCC with GP and then independently with patients they already treat in DMPs. **D** GPs expected these patients to benefit from an EPCC, i.e. patients with polypharmacy, patients without any diagnosis eligible for a DMP, “frequent attenders” (i.e. patients with >31 face-to-face consultations, telephone consultations or home visits (CTV) in two years) and “low-attenders”(less than 10 CTV in two years). **E **SeeFig. 1 (Step 3)

Thematic analysis of the co-creation process revealed five essential components of organising person-centred care in general practice. The following subheadings indicate the emerging essential components including steps 1, 2, and 3, and two overarching components. Figure 2 shows an overview of the co-creation process (per cycle: the facilitators, challenges and follow-up actions for the next cycle) including one exploratory phase and four act-observe-reflect-adjust cycles.

### Appropriate patient selection (step 1)

GPs mentioned that the added value of the EPCC did not always outweigh their invested time and efforts (cycle 1). GPs noticed that the formulated target group, namely patients with three or more chronic conditions, was quite large. They considered it unnecessary for GPs to invite all these patients for an EPCC. In addition, patients themselves sometimes did not see the added value of the EPCC. After having performed multiple EPCCs, GPs noticed that patients often did not feel chronically ill, despite having multiple documented chronic conditions.

*"We, as doctors, see it as disease burden because they use these specific medications, but if you turn it around and look at disease burden and consider this person with all these conditions, well, some people don't see themselves as chronic patients at all, but we see them as chronic patients.” (Cycle 1, focus group 2, GP16)*

*"Well, that whole conversation didn't seem relevant to me, I felt like, 'I don't know why you want to know,' that's what I said. I'm simply content, and everything is going well for me." (Cycle 1, patient interview GP10)*

GPs suggested that they should target those patients who may benefit most, which could encompass improved patient health outcomes and practice advantages (e.g. reduced unnecessary contact with the general practice). To optimise diversity in the selection of persons with multimorbidity, GPs suggested clustering certain chronic conditions, such as cardiovascular risks and diseases, asthma and COPD, and similar osteoarthritis conditions. The application of clustering yielded a total N of 3311 patients, whereas without clustering, the number was 4090. Also, to support differentiated patient selection, GPs received preselected patient lists where patients were grouped based on information extracted from the EMR (cycle 1 & 2). In the second cycle, these preselected lists were compiled based on the patient’s number of involved DMPs (0, 1 or more) and contact frequency with general practice (low, medium and high care). However, the co-creation team was encouraged by health insurers' representatives to contemplate beyond DMP care. In the third cycle, these pre-selected patient groups were defined as those for whom the GPs expected benefit from an EPCC. These concerned patients with polypharmacy, patients without any diagnosis eligible for a DMP, “frequent attenders” (i.e. patients with >31 face-to-face consultations, telephone consultations or home visits (CTV) in two years) and “low-attenders”(less than 10 CTV in two years). Other proposed patient groups that the researchers were not able to include in the selection report were complex newly registered patients and patients with low self-management capabilities.

Also, descriptive EMR data analysis was shared with the participating GPs. These revealed that approximately nine out of ten patients (before clustering) had at least one diagnosis eligible for a DMP. GPs suggested that a more person-centred approach could be incorporated in the already established DMP check-ups by PNs (see theme 2 ‘Division of tasks, roles and cooperation between GP and PN’).

*"Those who are already in a DMP should already be known. These existing consultations can be approached differently. For example, like GP_10. Yearly diabetes consultation: make it more person-centred. Look beyond just Diabetes." (Cycle 1, Research project weekly minutes)*

GPs mentioned that more insight into their own practice population (in the form of a descriptive analysis of EMR data) with multiple chronic conditions might help define the target definition. GPs were able to assess each patient’s anticipated benefit for an EPCC manually using their clinical judgement and filter out those who they wanted to invite first. Moreover, GPs suggested asking patients themselves whether they perceived added value in an EPCC. However, as both were expected to be time-consuming, researchers proposed to develop a prioritisation tool using EMR data (cycle 4). Such a tool could save GPs a lot of time and better target their efforts to those who need it most.

*"Yes, it would be good if we could better understand the numbers, for example, for the group that visits frequently: who are they, whom do they see, and so on? And I think it's also good to consider; are they already in a DMP or not? It would be good to categorise them more based on complexity and burden or vulnerability." (Cycle 1, focus group 2, GP16b)*

*In total, of all the people I have spoken with, most of them were not experiencing many problems, but there were a few people who indeed had areas that needed improvement. If you could identify those in advance with a sort of triage, and thus mainly see those people, that would, of course, be good." (cycle 3, focus group 2 GP22l)*

### Extended person-centred consultation (Step 2)

An EPCC in this pilot lasted on average 25 minutes, ranging between 20 and 40 minutes. GPs and PNs both mentioned that the EPCC positively impacted their job satisfaction (cycles 1 & 3, quantitative data analysis not shown). Also, GPs, as well as patients experienced an improved the doctor-patient relationship (cycle 1). The EPCC sometimes resulted in GPs gaining more insight into their patient's context and history, which benefitted future consultations (cycle 1). GPs expected that this time investment would lead to more efficient care in the long run. Patients mentioned they appreciated having more time with their GP, felt less rushed and felt heard (cycle 1).

*"Well, because you simply take the time for each other and are consciously engaged with one another, I think that automatically creates a deeper bond." (Cycle 2, patient interview GP12)*

Both GPs and PNs found the preparation and registration of EPCCs time-consuming. GPs sometimes struggled to keep the conversation medically substantive. GPs (cycle 1) and PNs (cycle 3) expressed the need for a conversation guide to help structure the conversation. To address this expressed need, the co-creation team developed two separate conversation guides (Supplementary box 2 and 3). To encourage patients to prepare for the consultations, some GPs used practical tools such as, Huber’s spider web with dimensions of positive health [22] and the Dutch Diabetes Foundation conversation card [23]. Experiences with these tools were mixed. The Diabetes conversation card for instance was regarded childish by some, however GPs felt it helped patients to prioritise which topics they wanted to discuss.

### Setting up personalised agreements and follow-up based on EPCC (Step 3)

GPs experienced shaping follow-up after an EPCC as challenging (cycle 2). When compared to structurally planned follow-up appointments in DMPs, individually tailored follow-up required more effort.

*"Yes. It does require more energy, I think, to provide personalised care than structured DMP care …The structure of DMP care normally offers a lot of support. It is often does provide stability. You can easily monitor them.. " (Cycle 4, focus group 3, GP20)*

GPs felt that shaping follow-up depended on the patient's self-management abilities. Patients with strong self-management capabilities could be expected to take more responsibility for their own health and care compared to patients with weak self-management capabilities. Three typical patient categories were characterised regarding their level of self-management ability and disease complexity, namely ‘self-reliant’, ‘support needed’ and ‘intensive support needed’ patients (cycle 2) (see Fig. 1*,* step 3). The EPCC could be used to assess and discuss a patient’s self-management capability, which could inform the frequency of follow-up appointments and allocation of responsibilities.

*We have a lot of vulnerable elderly patients, for whom it's often an assessment of whether they are still self-reliant or not. When we have doubts about that, I just schedule more regular check-up moments, and I also send my PN under the pretext of 'she's coming to measure the blood pressure,' and the PN sees how things are going there. I think it’s very important  to assess self-reliance." (Cycle 4, focus group 3, GP17w)*

Person-centred goalsetting was also seen as a potential way to individually tailor follow-up (cycle 2). One GP felt that the person-centred goals should be leading in shaping follow-up. GPs found it challenging to set person-centred goals with patients (cycle 2). They reported that patients found it difficult to formulate goals themselves. One GP suggested that patients are not used to take care of their own health. Also, doubts existed about the added value of setting person-centred goals: "the consultation is already a goal in itself" GPs felt. PNs indicated less difficulty in setting person-centred goals. They were already used to setting goals within DMPs. However, they found it challenging to assign responsibility for achieving the goals to the patient. They expressed a sense of responsibility for evaluating and fulfilling the goals that had been established. Both PNs and GPs found it difficult to retrieve the agreed goals in the EMR, which hampered revisiting them at follow up. Patients themselves were often not aware of the person-centred goal setting during the EPCC. In addition, analyses of the EMR journal reports showed that in 54 (23.6%) of the 229 extracted journal reports, goals were explicitly written down in the EMR. In 77 (33.6%) EPCCs, implicit goals were (also) noted. Table S2 demonstrates different categories of person-centred goals (e.g. lifestyle, social) with exemplary citations. The co-creation team inferred from these results that person-centred goals might have been discussed, but were not properly noted in the EMR. From a research perspective, it was not deemed surprising that GPs perceived person-centred goalsetting as challenging. Lack of training, experience and the current set up/structure of the information system (EMR) were mentioned as hampering factors in person-centred goalsetting. As a result, it might have been unclear for GPs what person-centred goalsetting entailed. Analysis of the goal setting assignments showed that some GPs mixed care agreement with person-centred goals (cycle 2), e.g., referral to physiotherapist instead of increasing mobility). Furthermore, one researcher mentioned that incorporating person-centred goal setting in the EPCC might have been too ambitious. Talking to patients about how to design their chronic care in order to address their needs and making the corresponding care agreements was considered challenging. Also, some organisational aspects might have contributed to difficulties in setting person-centred goals. For example, as EMRs are organised to record actions for specific diseases, no logical place was present to record disease-transcending care agreements and goals in the case of multimorbidity (cycle 4).

### Overarching components

#### Division of tasks, roles and collaboration between GP and PN

An overarching component related to all three steps is the division of tasks, roles and responsibilities: in step 1 with respect to prioritising multimorbid patients for EPCCs (by GP, PN or both), in step 2 related to the EPCC itself (by GP, PN or both) and in step 3 with respect to the execution of the agreements based on the EPCC (by GP, PN and patient). In the exploratory phase, GPs were convinced that the EPCC should be performed by the GP. However, since about nine out of ten patients were assumed to be treated within DMPs, in which PNs play a central role, it seemed only natural to explore perspectives on the role of PNs in the person-centred chronic care approach (cycle 2). GPs mentioned that PNs could assess whether the patient required an EPCC with the GP during routine DMP check-ups. GPs also stated that PNs could engage in person-centred conversations with specific patients (cycle 3) and signal existing problems in daily functioning including social, psychological or physical problems. These problems may not be exclusively related to conditions familiar to PNs.

*"You could also give the practice nurse more of a signalling role, so that she doesn't treat or address everything that comes up, but instead asks a number of targeted questions.” (Cycle 2, focus group 2, GP 15)*

GPs acknowledged that PNs should possess or develop certain skills and competences in order to deliver person-centred chronic care. GP noted a huge inter-PN variation in medical education (e.g. emergency room nurse or general practice secretary), work experience and workstyle (e.g. some PNs already had a person-centred role to some extent) (cycle 3). This variation should be considered when defining role division and responsibilities between GP and PN (cycle 3). PNs expressed the need for intervision to share experiences and discuss how to deal with patient questions about chronic conditions they know little about (cycle 3). Also, both GPs and PNs stressed the added value of scheduling time to prepare and/or debrief EPCCs together to promote the signalling role of the PN and to organize person-centred follow-up responsibilities. Since this time was not always planned structurally, GPs and PNs were encouraged to do so in the next cycle (cycle 3).

*If I don't know something, I consult with the GP. Then we'll examine the issue together. You learn from that, and then you become a bit wiser. The next time, you know it. Yes, I think it's also a matter of practice, indeed." (Cycle 3, focus group 1, GP16_PN)*

### Sustainable organisation

In the fourth cycle, participating GPs and PNs were encouraged by the researchers to formulate several preconditions for successfully achieving sustainability. First and most importantly, GPs and PNs found that appropriate remuneration is needed to cover the additional time commitment in organising, preparing and conducting the EPCC.

*"If we really say, 'we want to make more time for a specific group, to truly provide personalised care,' then we need more facilities, time, and money for that.” (Cycle 4, focus group 2, GP16k)*

Second, an EMR that facilitates registration of disease-transcending information instead of disease-specific, is essential. Ideally, a person-centred plan per patient should be available (covering all conditions), in which every involved healthcare provider can see the agreements and actions requested. Third, training in delivering person-centred chronic care, person-centred communication and goalsetting should be offered to all general practice care providers (and trainees). For successful implementation, this approach should be a practice-wide initiative. Fourth, a prioritisation tool would be very welcome to support GPs who to invite first for an EPCC based on anticipated benefit. Ideally, such a priorisation tool should be based on an automated algorithm that can run in the EMR. Such a tool may potentially save GPs time and better target comprehensive efforts. Fifth, a scheduling procedure was advocated to plan EPCCs and to invite patients in advance in a timely manner. Also, GPs mentioned that scheduling EPCCs on a fixed day and time increased feasibility (cycle 1).

*"It took a lot of time. Especially how I did it, with double notes; every medical aspect that was discussed, I noted it in the EMR and linked it to a specific diagnosis. (Cycle 1, focus group 2, GP15)*

Last, to facilitate the adoption of the chronic care approach among affiliated general practices within the care cooperation, it was proposed by GPs that designated 'local clinical champions' be appointed to effectively disseminate and embed its principles.

## Discussion

### Summary

A person-centred chronic care approach targeting patients with multimorbidity in general practice was developed in co-creation with stakeholders. Essential elements of this approach included (step 1) appropriate patient selection for (step 2) an extended person-centred consultation, and (step 3) personalised agreements and follow-up. The main facilitating factors were the better therapeutic relationship GPs experienced with their patients, the positive effect on their job satisfaction and the patients appreciating having more time with their GP, feeling heard and less rushed. The approach requires a deliberate division of tasks, roles and collaboration between GP and PN tailored to the patient, the local setting and care personnel. Elicited challenges for implementation include a prioritisation tool to support GPs appropriately who to invite first for an extended consultation, appropriate remuneration and time to conduct the extended consultations, training in delivering person-centred chronic care available for all general practice care providers and an electronic medical record system that facilitates registration of information that transcends separate diseases. Designated ‘local clinical champions’ seem key to effectively disseminate and embed its principles.

### Strengths and limitations

A major strength of the PAR approach was the involvement of local stakeholders in every step of the co-creation process, leading to ownership of the project. In addition, the flexible nature of this type of research allowed for adjustments to be made, enabling piloting of learned lessons. Another strength of this study was the clear distinction made between financial and substantive aspects of the person-centred approach that promoted thinking beyond financial constraints during the co-creation process.

One unavoidable limitation of this study was that the approach was developed while the DMPs and their structurally planned follow-up and administrative obligations were still in place. Another unforeseen event was the COVID-19 pandemic that broke out halfway through the study, leaving participants with less time and attention for the project. Despite this, the pandemic also gave unprecedented opportunities to draw lessons from the sudden suspension of care delivered within DMPs and it provided an opportunity for researchers and participants to think outside the boundaries of this type of care. Emerging questions were, for example, "which patients do I need to keep in my focus as a GP, now that DMP care has been disrupted?” and “does digital healthcare (e.g., e-consultations) provide new possibilities for monitoring patient well-being, offering an alternative approach to the traditional in-person visits for standard check-ups at the medical practice?”. Another limitation might have been that GPs and their PNs who participated in this study, were intrinsically motivated and may not fully represent their colleagues. Underrepresentation of less motivated GPs and PNs needs to be taken into account as this may pose an additional challenge for dissemination.

### Comparison to existing literature

To our knowledge, this is the first study that developed a person-centred care approach in general practice for patients with multimorbidity in a bottom-up manner by applying PAR. Earlier studies in similar healthcare systems reported limited and inconclusive evidence on the potential benefits and risks of such a person-centred care approach. In addressing potential contributors to the 'inconclusive' evidence, Smits et al., and the UK Medical Research Council Guidance emphasize the careful development and refinement of complex interventions, such as through PAR, as implemented in the current study [12, 14]. Furthermore, Salisbury et al. conducted a pragmatic cluster-randomised trial in general practice that aimed to implement and evaluate a patient-centred intervention to improve the management of patients with three or more chronic diseases. The authors found no significant effects on patients' health-related quality of life [13]. The process evaluation of this trial demonstrated implementation difficulties. In addition, the authors suggested potential improvements, including appropriate patient selection, preparation of patients, incorporating skills practice in the training (i.e., agenda setting and collaborative action planning with the patient) and flexibility to tailor follow- intensity to patient need. The PAR approach of the current study, however, allowed to address some of these aspects in more detail and from an early stage of the project, such as identifying the appropriate patient group for the EPCC by GPs. During the research cycles, adjustments were made to increase diversity in the selection of persons with multimorbidity. Additionally, it seems that the Medical Research Council's Framework for Developing and Evaluating Complex Interventions does not explicitly consider the identification of the most appropriate patient group during intervention development [14]. Given the results of this study, this aspect may warrant further attention. Lastly, Smits et al. highlight the challenge of defining outcomes applicable to various combinations of diseases in multimorbidity [12]. They propose examining outcomes like goal attainment and self-management capabilities, which have been explored in the present study.

While the focus of this study was on general practice care, the authors acknowledge the importance of person-centred care in collaboration with other primary or community care providers (such as paramedics and the social domain), and specialised care providers. Additional key components of integrated care for multimorbidity have been identified by initiatives such as the SELFIE study, the EU Joint Action on Chronic Diseases and Healthy Ageing across the Life Cycle (JA-CHRODIS) and Raaijmakers et al., based on the literature and through international expert meetings of stakeholders [2, 24, 25]. Components such as information exchange, development of a system to consult experts outside the core team, and involvement of the informal social network, were mentioned. Although not addressed in this study, exploring these components could be done in future (participatory action) research. Nevertheless, by focusing on a specific aspect of care delivery (i.e. care management in general practice), the present study was able to make meaningful progress by taking small, but crucial steps towards successful implementation.

### Reflexive insight into the researcher's role in the co-creation process

Researchers adopted dual roles in the PAR approach, with a senior researcher acting as a participating GP and project initiator. Recognizing the dual nature of these roles (objective researcher and participating GP), close attention was paid to clearly delineating these roles during meetings. The other two senior researchers monitored this process, maintaining an appropriate distance from the subject matter, ensuring a balanced dynamic within the research group. The researchers' influence was also notable in advocating for person-centred goalsetting, seen as potential means for tailoring follow-up and measuring complex personalized approaches' effectiveness. Despite their emphasis, this did not manifest in GP discussions, raising doubts about its perceived value. Additionally, researchers influenced the co-creation process by advocating for a limited number of participating general practices, contrasting the care group's desire for early expansion to enhance project ownership.

### Implications for research and practice

The main challenge in this study was defining the target group: those multimorbid patients who are expected to benefit most from person-centred needs assessment and care coordination with their GP. More research is needed to define this target population more specifically. To successfully implement this approach in daily practice, critical preconditions must be adequately addressed. Most importantly, without sufficient time and appropriate remuneration for this expanding patient group, its realisation will remain unattainable. Also, patient selection (tools) and an EMR that facilitates disease-transcending registration of information relevant for personalised care should also be addressed. To encourage the widespread adoption of this chronic care approach, training in delivering person-centred chronic care should be offered to all general practice care providers, and designated local clinical champions should be appointed to effectively disseminate and embed its principles. Future research should evaluate its effectiveness on ‘Quadruple aims’ both short and long term.

## Conclusion

A person-centred chronic care approach targeting patients with multimorbidity in general practice was developed in co-creation with stakeholders. Challenges and facilitators were identified. Challenges need to be tackled before widespread implementation. Future evaluation on the quadruple aims is recommended.

### Supplementary Information

**Supplementary Material 1. **

## Data Availability

For privacy reasons the data cannot be made publicly available.

## Abbreviations

GP: General practitioner
HZK: ‘Huisartsen Coöperatie Zuid Kennemerland’
ICPC: International Classification of Primary Care
EPCC: Extended person-centred consultation
PN: Practice nurse
