# Supplementary material for: Development of a person-centred care approach for persons with chronic multimorbidity in general practice by means of participatory action research
Source: BMC Prim Care. 2024 Apr 16;25:114. doi: 10.1186/s12875-024-02364-x (PMC11020638; doi:10.1186/s12875-024-02364-x)
Supplement: Supplementary file 1 — Supplementary Material 1. [file 12875_2024_2364_MOESM1_ESM.docx]

# **Supplementary data**

**
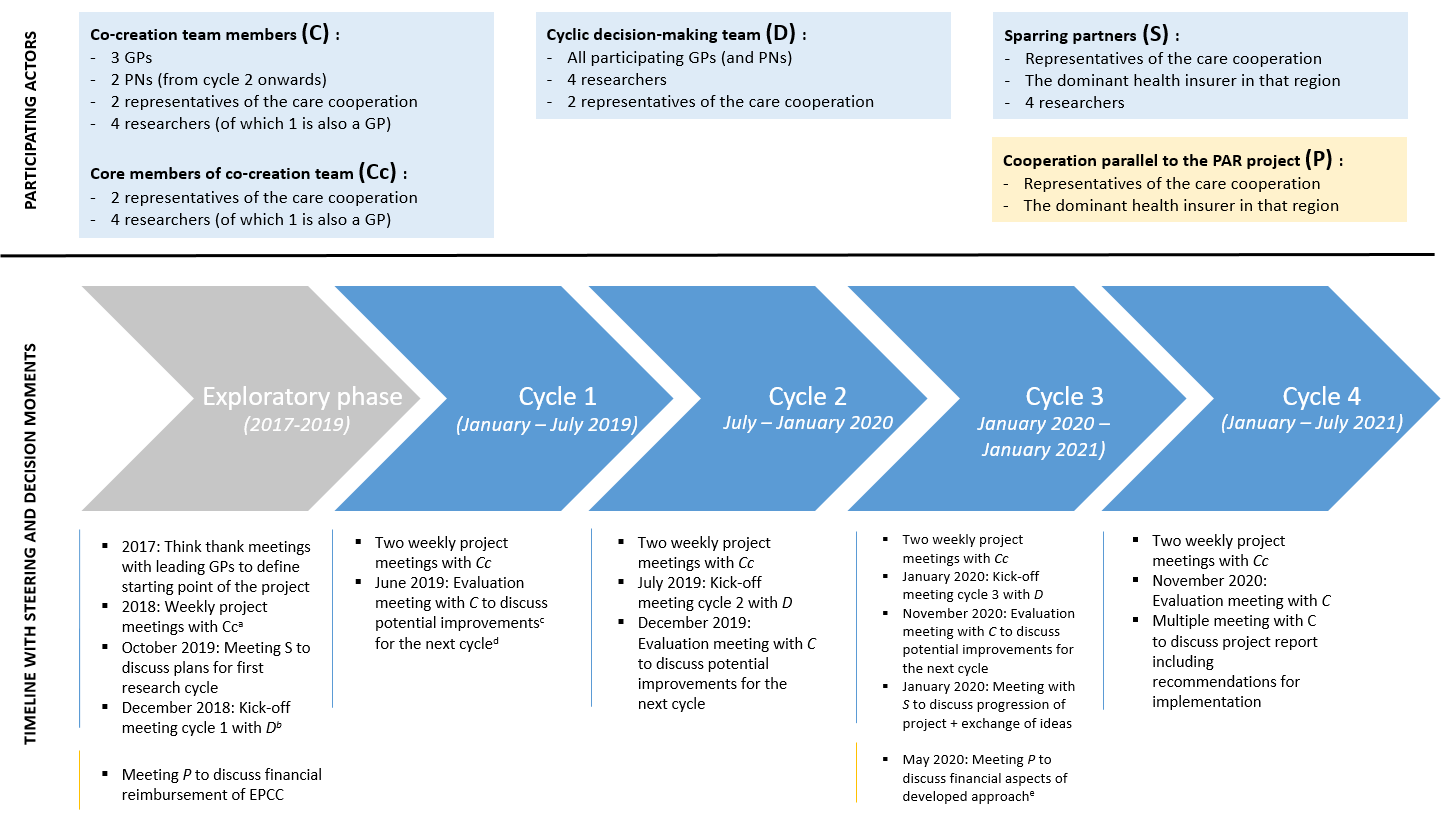
**

*Figure S1*: Visualisation of the steering and decision moments (including the involved actors) during the co-creation process.

*^a^ To discuss process and content aspects of the project, ^b^ based on all gathered information (e.g. focus groups, interviews, analysis of EMR data) concerning the action phase., ^c^ The facilitators, challenges and potential adjustments for the next research cycle are discussed. The team decides upon the follow-up actions for the next cycle. , ^d^ This meeting is preparatory to the kick-off meeting of that cycle. ^e^ Collaboration between care cooperation and health insurer parallel to the PAR project to discuss financial aspects during transition period (disease management to person-centred management)*

**Table S1: List of chronic conditions^a^**

| **Clusters** | ***Conditions^b,c^*** | ***ICPC-code(s)*** |
| --- | --- | --- |
| HIV/Aids | HIV/Aids | B90 |
| Cancer | All malignant cancer types | A79, B72-B72.01-B72.02 , B73, D74, D75, D76, D77-77.01- D77.02-D77.03-D77.04, L71-L71.01, N74, R84, R85, S77-S77.01-S77.02-S77.03-S77.04, T71, U75, U76, U77, W72, X75, X76-X76.01, X77-X77.01-X77.02, Y77, Y78-Y78.01-Y78.02-Y78.03 |
| Bowel disorders | Diverticular disease | D92 |
|  | Crohn disease | D94 |
|  | Ulcerative colitis | D94 |
| Cardiovascular | Congenital heart disease | K73-K73.01-K73.02 |
| (i.e. DMP) |  |  |
|  | Infectious disease of heart and/or blood vessels | K70 |
|  | Acute rheumatoid heart disease | K71-K71.01-K71.02 |
|  | Non-rheumatic Valvular Heart Disease | K83-K83.01-K83.02 |
|  | Heart failure | K77-K77.01-K77.02 |
|  | Angina Pectoris | K74-K74.01-K74.02 |
|  | Acute myocardial infarction | K75 |
|  | Atrial fibrillation/flutter | K78 |
|  | Hypertension | K86, K87 |
|  | Transient Ischemic Attack (TIA) | K89 |
|  | Cerebrovascular accident (CVA) | K90 |
|  | Intermittent claudication | K92 |
|  | Aneurysm aortae | K99 |
|  | Hypercholesterolemia | T93 |
| Musculoskeletal | Fibromyalgia | L18, L18.01 |
|  | Rheumatoid arthritis | L88-L88.01-L88.02 |
|  | Cox arthrosis | L89 |
|  | Gon arthrosis | L90 |
|  | Other arthrosis | L91 |
|  | Cervical spine syndromes | L83-L83.01 |
|  | Osteoarthritis spondylosis of the spine | L84-L84.01-L84.02 |
|  | Low back pain with radiation | L86-L86.01 |
|  | Osteoporosis | L95-L95.02-L95.02 |
| Neurologic | Multiple sclerosis (MS) | N86 |
|  | Parkinson's disease | N87-N87.01 |
|  | Epilepsy | N88 |
|  | Migraine | N89 |
|  | Cluster headache | N90 |
|  | Trigeminal neuralgia | N92 |
|  | Other neuropathies | N94 |
| Alcohol abuse | Chronic alcohol abuse | P15 |
| Psychological | Sleeping disorder | P06 |
|  | Schizophrenia | P72 |
|  | Affective psychosis | P73-P73.02 |
|  | Depression | P76-P76.01 |
|  | Anxiety disorder | P74-P74.01-P74.02 |
|  | Personality disorder | P80-P80.01-P80.02 |
| Respiratory (i.e. DMP) | Chronic Obstructive Pulmonary Disease (COPD) | R95 |
|  | Asthma | R96-R69.01-R69.02 |
|  | Chronic bronchitis | R91-R91.01-R91.02 |
| Thyroid | Persistent thyroglossal duct/cyst | T78 |
|  | Benign neoplasms of thyroid gland | T72 |
|  | Hyperthyroidism | T85 |
|  | Hypothyroidism | T86 |
| Diabetes Mellitus (i.e. DMP) | Diabetes Mellitus type I | T90.01 |
|  | Diabetes Mellitus type II | T90.02 |
| Urinary | Kidney disease | U99 |
| Psoriasis | Psoriasis with methotrexate use | S91 |
| Obesity | Adiposity | T83 |
| Smoking | Tobacco abuse | P17 |
| Eye disease | Macular degeneration | F84 |

^a^ The criterion ≥ 3 chronic diagnoses was formulated after considering estimations of eligible patients based on routine care records of three local practices who are part of Academisch Netwerk Huisartsengeneeskunde (ANH). Consequently, a manageable amount of patients to participate in this study was achieved. Moreover, since this criterion was applied in an earlier study in a comparable setting on this topic (1), we chose to stay in line with existing literature and therefore formulated this criterion.

^b^ This list ‘chronic conditions’ was developed in the participatory action research (COPILOT) project (2) (where this study was part of), using multiple brainstorm sessions with a group of GPs. Consensus was reached after comparing the developed list with existing lists of chronic conditions (3).

^c^ Definition: ever diagnosed and ≥ 1 contact in the 2 observation years. For the diagnoses cancer, HIV/Aids, Crohn, Ulcerative colitis and schizophrenia the definition ‘diagnosed and ≥1 contact ever’ was used. Contacts included all types of contacts, such as consultations, visits, repeat prescriptions and consultations by phone and by e-mail.

**Box S1:** *Work satisfaction questionnaires for general practitioners and practice nurses*

**Questionnaire for General Practitioner**

**General information:**

1. **Gender:** Male/ Female
2. **Age:** … years
3. **Type of practice:**

single-handed practice Duo practice Group practice

1. **How long have you been working at this practice?**…. years

**Statements:**

The following statements relate to the care for patients with multiple chronic diseases simultaneously. On a scale of 1-10, please indicate the extent to which you agree with each statement.

**Statement 1**: I find satisfaction in my duties related to providing chronic care to patients with multiple chronic diseases.

0 1 2 3 4 5 6 7 8 9 10

**Statement 2**: I am content with the collaboration with the practice nurse during my duties related to providing chronic care to patients with multiple chronic diseases.

0 1 2 3 4 5 6 7 8 9 10

**Statement 3:** The care for patients with multiple chronic diseases **within** disease management programmes is burdened with administrative tasks that do not benefit the patient.

0 1 2 3 4 5 6 7 8 9 10

**Statement 4:** Administrative obligations within my duties related to providing chronic care to patients with multiple chronic diseases **within** disease management programmes are minimized to the necessary extent.

0 1 2 3 4 5 6 7 8 9 10

**Statement 5:** The current chronic care protocols for patients with multiple chronic diseases treated **within** disease management programmes significantly influence the content and frequency of consultations.

0 1 2 3 4 5 6 7 8 9 10

**Statement 6:** I find satisfaction in working with standardized protocols in my duties related to providing chronic care to patients with multiple chronic diseases **within** disease management programmes.

0 1 2 3 4 5 6 7 8 9 10

**Statement 5:** I am content with the quality of care I provide to patients with multiple chronic diseases **within** disease management programmes.

0 1 2 3 4 5 6 7 8 9 10

**Statement 6:** I am content with the quality of care I provide to patients with multiple chronic diseases **who are not treated within** disease management programmes.

0 1 2 3 4 5 6 7 8 9 10

**Statement 7:** I provide tailored care to patients with multiple chronic diseases **within** disease management programmes.

0 1 2 3 4 5 6 7 8 9 10

**Statement 8:** I provide tailored care to patients with multiple chronic diseases **who are not treated within** disease management programmes.

0 1 2 3 4 5 6 7 8 9 10

**Statement 9:** I proactively approach patients with multiple chronic diseases **within** disease management programmes.

0 1 2 3 4 5 6 7 8 9 10

**Statement 10:** I proactively approach patients with multiple chronic diseases **who are not treated within** disease management programmes.

0 1 2 3 4 5 6 7 8 9 10

**Statement 11:** I believe I have sufficient time for patients with multiple chronic diseases **within** disease management programmes.

0 1 2 3 4 5 6 7 8 9 10

**Statement 12:** I believe I have sufficient time for patients with multiple chronic diseases **who are not treated within** disease management programmes.

0 1 2 3 4 5 6 7 8 9 10

**Elaboration:**

**Box S1:** **Questionnaire for practice nurses**

**General information:**

1. **Gender:** Male/ Female
2. **Age:** … years
3. **Type of practice:**

single-handed practice Duo practice Group practice

1. **How long have you been working at this practice?**…. years

**Statements:**

The following statements relate to the care for patients with multiple chronic diseases simultaneously. On a scale of 1-10, please indicate the extent to which you agree with each statement.

**Statement 1**: I find satisfaction in my duties related to providing chronic care to patients with multiple chronic diseases.

0 1 2 3 4 5 6 7 8 9 10

**Statement 2**: I am content with the collaboration with the general practitioner during my duties related to providing chronic care to patients with multiple chronic diseases.

0 1 2 3 4 5 6 7 8 9 10

**Statement 3:** The care for patients with multiple chronic diseases within disease management programmes is burdened with administrative tasks that do not benefit the patient.

0 1 2 3 4 5 6 7 8 9 10

**Statement 4:** Administrative obligations within my duties related to providing chronic care to patients with multiple chronic diseases within disease management programmes are minimized to the necessary extent.

0 1 2 3 4 5 6 7 8 9 10

**Statement 5:** The current chronic care protocols for patients with multiple chronic diseases treated within disease management programmes significantly influence the content and frequency of consultations.

0 1 2 3 4 5 6 7 8 9 10

**Statement 6:** I find satisfaction in working with standardized protocols in my duties related to providing chronic care to patients with multiple chronic diseases within disease management programmes.

0 1 2 3 4 5 6 7 8 9 10

**Statement 7:** I am content with the quality of care I provide to patients with multiple chronic diseases within disease management programmes.

0 1 2 3 4 5 6 7 8 9 10

**Statement 8:** I provide tailored care to patients with multiple chronic diseases within disease management programmes.

0 1 2 3 4 5 6 7 8 9 10

**Statement 9:** I proactively approach patients with multiple chronic diseases within disease management programmes.

0 1 2 3 4 5 6 7 8 9 10

**Statement 10:** I believe I have sufficient time for patients with multiple chronic diseases within disease management programmes.

0 1 2 3 4 5 6 7 8 9 10

**Elaboration:**

**Box S2:** **Guide Extended person-centred consultation**

The broad chronic care consultation gives the GP and the patient the opportunity to discuss the patient's entire health situation.

To ensure that all chronic care issues are addressed and there is sufficient time to discuss them in depth together, it is important to provide a clear structure to the form and content of the conversation.

The consultation is further elaborated below.

**Patient input**

- Discuss the (overall) current situation of the patient:
- chronic conditions
- Medications
- mental state
- family and other circumstances that influence symptoms and perceptions
- what agreements are ongoing with other healthcare providers
- Then zoom in on the (chronic) care aspects:
- what does the patient consider important to address in relation to his/her health,
- what is the desired situation compared to the current one, what does the patient want to achieve?
- Check whether there is motivation for change:
- what would the patient most like to change and why is that important?
- which change is feasible, in what timeframe?

**Input GP / PN**

- Give direction to the conversation, paying attention to circumstances, but ensure that the consultation remains sufficiently medical and healthcare related . At the end of this broad chronic consultation, all points of interest should have been discussed with the patient.
- Provide information relevant to the patient's situation about:
- existing chronic conditions
- possibilities and possibly expected outcomes of change/adaptation
- risks of paying less or no attention to a certain situation/symptoms

**Shared decision-making between patient and GP (and possibly PN)**

- Based on the patient's motivation/needs, discuss the treatment plans and options, with pros and cons.
- Make a choice for treatment (priorities), follow-up appointments or referral. Agree what the patient will work on (objective, term) and what the GP and/or PN will do.
- Agree when the next consultation will take place; when would you like to evaluate together how things have gone in the past period (long-term agreement).
- Record the agreements made (briefly) in the EMR (code A58) and in a patient letter. Give the printed letter to the patient. During the consultation, we will examine together whether the agreements have been fulfilled and which objectives have been achieved.

**Assessment after the EPCC**

After the EPCC, make an assessment of the patient sitting opposite you and record this in the EMR. You can divide the patient into 3 groups:

**Self-reliant: high degree of independence**

For example:

• GP considers monitoring by PN sufficient (if involved)

• Is appropriate and adequately monitored elsewhere (hospital)

• Patient can adequately signal when health changes

• Patients > 5 years in DMP and stable

• Non-DMP conditions in stable phase

**Support needed: patient's structural indicated care needs during the year**

For example:

• GP sees importance in periodic check-ups

• The GP believes guidance from PN is sufficient

• Diabetes insulin dependent

• COPD heavier disease burden

• Non-DMP conditions with moderate/high disease burden

**Intensive support needed: patient's structural complex care needs throughout the year**

For example:

• GP sees importance in periodic check-ups

• The GP believes guidance from GP is necessary

• The GP believes that guidance from several care providers is necessary

• Multimorbidity with heavy disease burden

• Serious psychosocial problems

**Box S3: Extra guide for PNs**

v

**Record (2 options)**:

- Entire consultation under code A58 with episodic text 'copilot'.
- Per condition (S and P lines) + note ‘EPCC’ for one condition

**Discuss:**

- Debrief each patient with the GP (e.g. one set time during the week).
- Go through chronic episodes.
- Read information on conditions if necessary: Home doctor patient letters.
- Think about what you want to know and/or measure (at least).
- Try to let the rest come from the patient (rather than finishing the protocol entirely).
- .
- Invite the patient by phone or invitation letter.
- Encourage preparation by patient (e.g. use Dutch Diabetes Federation discussion card, Machteld Huber, etc.).
- Explain reason for consultation and role of the PN.

*Your role is to signal and not necessarily solve.*

1. **Exploration**

- Start open:
- how are you/your health?
- Are there certain things you would like to discuss?
- What do you encounter most? What are you already doing about this yourself?
- Do you still need an explanation?
- Run through the list of chronic conditions:
- Do you have problems with this condition?
- Do you have any questions about the condition?
- What would you still like to improve? Where do you see opportunities? Are things holding you back?
- Setting personal goals:
- Do you think something should change regarding your chronic diseases / regarding disease X?
- How do you want to achieve this? How do you see our role (general practice) in this?

1. **Summarise**
2. **Tailor follow-up:**

**-**  Assess the patient’s self-management capabilities (note in EMR)

- Regarding person-centred goals: Who does what? When do we evaluate whether we have succeeded?
- Appointments regarding check-ups: How do you experience the DMP checks? Too much, too little? Do you benefit from them? What will be the new frequency?

**Prepare**

**After the consultation**

**Consultvoering**

**Invite**

***Table S2****.* Categories of personal goals with exemplary citations.

| **Categories of formulated personal goals** | | **Citations** |
| --- | --- | --- |
| Explicit goals | Lifestyle goals | *“Goal: to improve fitness and thereby prevent hospitalization.” (medical record, study number 161285) “Final goal is to weigh 100kg, but he is not sure when.” (medical record, study number 111323)* |
|  | Social goals | *“Goal: 1 Restore contact with mother.” (medical record, study number 191253) “Goal: 3 Keep social isolation as reduced as possible.” (medical record, study number 191317)* |
|  | Mental health goals | *“Goal: 4 Does not want to live anymore: keep in touch.” (medical record, study number 191253) “Goals for this year: more processing and creating more mental space for themselves.” (medical record, study number 171412)* |
|  | Structured disease management goals | *“Goal: 1 Properly regulate diabetes mellitus 2 and monitor madam her ability to self-inject.” (medical record, study number 191317) “The aim is to get rid of metformin medication.” (medical record, study number 111116)* |
|  | Care agreement goals | *“Goals: analyse lung problems and treat them if necessary.” (medical record, study number 171016) “Goal: referral to an ENT specialist.” (medical record, study number 151051)* |
|  | Medical goals | *“Goal: Less wrist pain.” (medical record, study number 121062) “Goal: improving muscle strength around the left knee to reduce pain caused by arthrosis.” (medical record, study number 171178)* |
| Implicit goals | | *“Would like to use less medication, for example the cholesterol medication.”(medical record, study number 121219) “Patient would like to improve their fitness.”(medical record, study number 161216)* |

**References**

1. Salisbury C, Man MS, Bower P, Guthrie B, Chaplin K, Gaunt DM, et al. Management of multimorbidity using a patient-centred care model: a pragmatic cluster-randomised trial of the 3D approach. Lancet. 2018;392(10141):41-50.

2. Bogerd MJ, Slottje P, Schellevis FG, Giebels A, Rijken M, van Hout HP, Reinders ME. From protocolized to person-centered chronic care in general practice: study protocol of an action-based research project (COPILOT). Primary health care research & development. 2019;20.

3. O'Halloran J, Miller GC, Britt H. Defining chronic conditions for primary care with ICPC-2. Fam Pract. 2004;21(4):381-6.
